# Supplementary material for: Ultrahigh transverse thermoelectric power factor in flexible Weyl semimetal WTe2
Source: Nat Commun. 2022 Jul 7;13:3909. doi: 10.1038/s41467-022-31372-7 (PMC9262886; doi:10.1038/s41467-022-31372-7)
Supplement: Supplementary file 1 — Supplementary Information [file 41467_2022_31372_MOESM1_ESM.pdf]

Supplementary Information

Ultrahigh transverse thermoelectric power factor in flexible Weyl semimetal WTe_2

Yu Pan[1,*], Bin He[1], Toni Helm[2], Dong Chen[1], Walter Schnelle[1], Claudia Felser[1,*]

[1]*Max Planck Institute for Chemical Physics of Solids, Nöthnitzer Str. 40, Dresden 01187, Germany.*
[2]*Dresden High Magnetic Field Laboratory (HLD-EMFL), Helmholtz-Zentrum Dresden-Rossendorf, Dresden 01328, Germany.*
E- mail address: Yu.Pan@cpfs.mpg.de; Claudia.Felser@cpfs.mpg.de

SEM and EDX results

The microstructure of the WTe_2 single crystal in scanning electron microscopy (SEM) are shown in Supplementary Fig. 1a. A ribbon-like single crystal is illustrated, in which the longest direction is along the a-axis, and the c-axis is perpendicular to the layer. The composition and homogeneity were examined by the energy-dispersive X-ray spectroscopy (EDX), as shown in Supplementary Fig. 1b. No impurity was found and the elemental ratio of W:Te is very close to 1:2, indicating the pure phase of WTe_2.

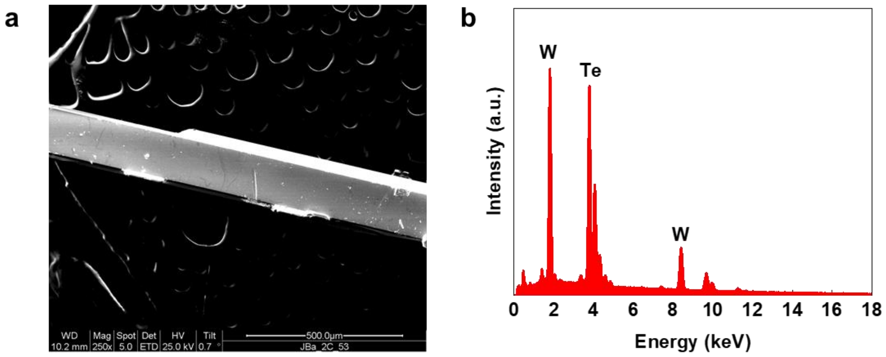

Supplementary Figure 1. Microstructure and composition. a Microstructure of the WTe_2 single crystal in scanning electron microscopy. **b** energy-dispersive X-ray spectroscopy.

Magnetoresistance under DC field up to 9 T

The magnetoresistance measured under DC field by PPMS from 2 K to 300 K are shown in Supplementary Fig. 2. Large MR values are observed, reaching 10^6 % at 2 K, 9 T. The obvious oscillation and the large MR demonstrate the high quality of the single crystals.

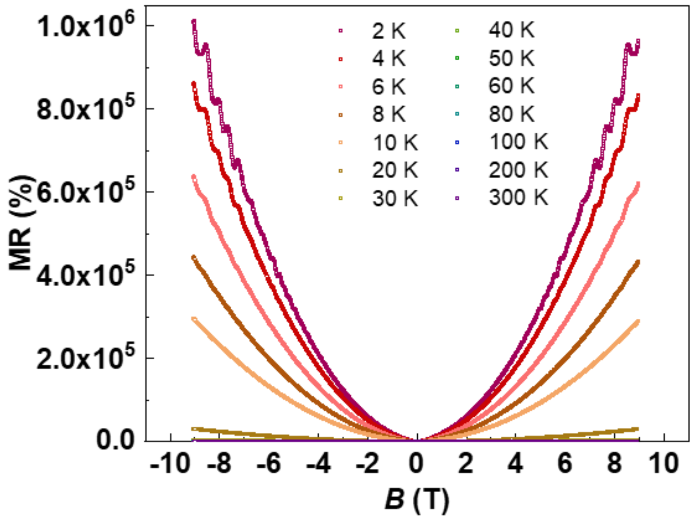

Supplementary Figure 2. Magnetoresistance. Magnetoresistance from 2 K to 300 K up to 9 T, measured with current applied along *a*-axis and magnetic field along *c*-axis.

Temperature dependence of resistivity

The WTe_2 single crystal shows a metallic transport behavior with a decreasing resistivity upon cooling, as shown in Supplementary Fig. 3. A large RRR value of 1572 is observed, demonstrating the high quality of the single crystal. Notably, the resistivity is very small particularly at low temperatures. Compared to the common thermoelectric semiconductors which show a resistivity at the order of mΩ cm, the resistivity of WTe_2 single crystal is much lower.

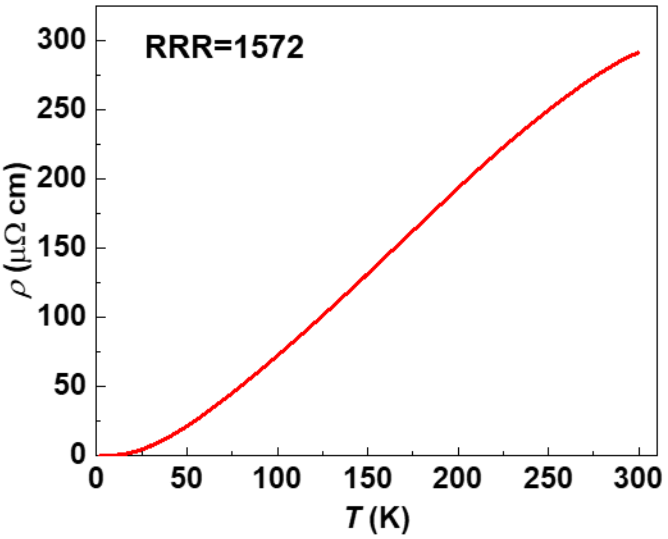

Supplementary Figure 3. Resistivity. Temperature dependence of longitudinal resistivity. An RRR value of 1572 is observed from the temperature dependence of the resistivity.

Bridgman relation

According to the Onsager reciprocal relation, the Ettingshausen coefficient P can be determined by the isothermal Nernst coefficient N via the Bridgman relation:

$$P_{xy} = N_{yx}T/\kappa_{xx}$$

In which κ is the thermal conductivity and T is the absolute temperature. Herein, the reported Nernst and Ettingshausen performances do not have to obey the above relation because: (a) the Nernst effect is measured under adiabatic condition instead of isothermal condition; (b) both the Ettingshausen and the Nernst effects are measured in the transverse direction (along b-axis) by applying the current/heat flux along the longitudinal direction (along a-axis), i.e. P_{yx} and N_{yx} (note that $P_{xy} \neq P_{yx}$ in WTe_2 due to its point group of mm2). Nevertheless, we roughly estimated P_{xy} using the adiabatic N_{yx}. Taking the data at 20.7 K as an example, $P_{xy} = N_{yx}T/\kappa_{xx} = 2\times10^{-5}$ $KA^{-1}m$, which is close to the measured P_{yx} at 23.1 K ($P_{yx} = (\nabla T_{yx}/j_{xx})/B_z = 0.6 \times10^{-5}$ $KA^{-1}m$).

Fitting curves of the Hall conductivity

Supplementary Fig. 4 shows the fitting curves of the Hall conductivity from 2 K to 50 K. The experimental results (black dots) are well fitted by the two-band model (red curves), according to which the concentration and mobility of electrons and holes are resolved.

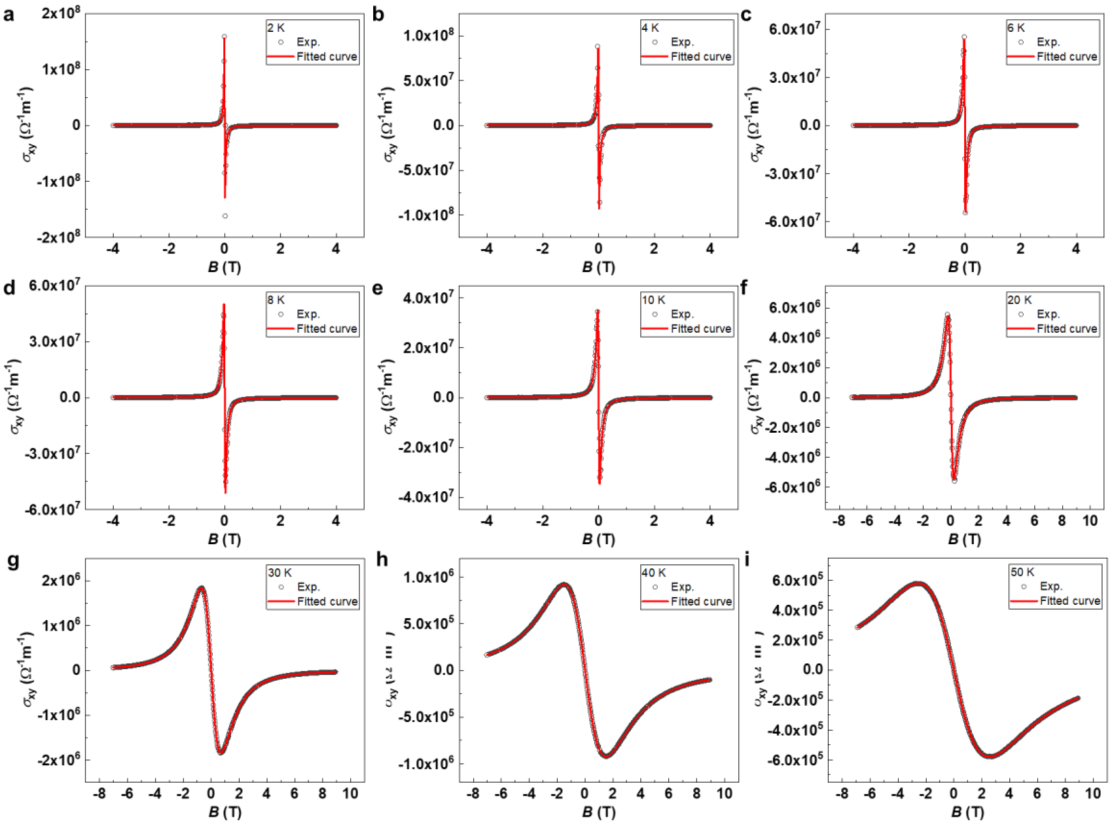

Supplementary Figure 4. Fitting curves of the Hall conductivity at different temperatures. **a** 2 K, **b** 4 K, **c** 6 K, **d** 8 K, **e** 10 K, **f** 20 K, **g** 30 K, **h** 40 K, **i** 50 K.

Assuming a spherical Fermi surface, we can roughly estimate the carrier concentration n from the SdH oscillation, $n = (1/3\pi^2)(2eF/\hbar)^{3/2}$, in which F is the frequency, e is the elementary charge, and $\hbar$ is the reduced Planck constant. Supplementary **Table 1** illustrates the results. For the total hole concentration, $n_h = 2\times(n_{h1} + n_{h2}) = 3.28E19\ cm^{-3}$, for the total electron concentration, $n_e = 2\times(n_{e1} + n_{e2}) = 3.64E19\ cm^{-3}$, considering that there are two sets of pockets in the Brillouin zone. The SdH estimated carrier concentration is close to the Hall results, within the ~10^{19} cm^{-3} order. Small differences can be due to the anisotropy of the Fermi surface.

Supplementary Table 1. Estimated carrier concentration from SdH oscillations.

Pockets	Frequency (T)	Carrier concentration (cm^{-3})
hole 1	94	0.51E19
electron 1	130	0.84E19
electron 2	144	0.98E19
hole 2	159	1.13E19

Thermoelectric performance

Given that longitudinal thermoelectrics based on Seebeck effect is extensively studied for the comprehensive thermoelectric performance, BiSb[1] and $(Bi,Sb)_2Te_3$,[2] which are the best longitudinal thermoelectric materials below and near 300 K, are respectively chosen here for the comparison. For example, WTe_2 yields a resistivity (at 10 K, 9 T) of ~1 mΩ cm, comparable to those of the state-of-the-art thermoelectric semiconductors including BiSb (at 100 K, 0 T) and $(Bi,Sb)_2Te_3$ (at 300 K, 0 T), as shown in Supplementary Fig. 5a.

Topological semimetals can have comparable or even higher *PF* values than the state-of-the-art longitudinal thermoelectric semiconductors. Moreover, topological semimetals are highly promising, showing the Nernst *zT* values at the same order as that of the state-of-the-art longitudinal thermoelectric semiconductors (Supplementary Fig. 5b). Herein, to calculate the Nernst *zT* of WTe_2, adiabatic Nernst thermopower, adiabatic thermal conductivity, and adiabatic resistivity are used. Using adiabatic resistivity, the obtained Nernst *zT* is the adiabatic figure of merit as defined in previous report.[3]

For practical application, both high *PF* and high *zT* are desired. Supplementary Fig. 5c summarizes the Nernst *PF* and *zT* cvalues of WTe_2 and other topological semimetals, including $ZrTe_5$[4] and Cd_3As_2,[5] with a comparison to the Seebeck *PF* and Seebeck *zT* values of the best longitudinal thermoelectric semiconductors, BiSb[1] and $(Bi,Sb)_2Te_3$.[2] These results suggest that topological semimetals are highly suitable as transverse thermoelectric materials, particularly at extremely low temperatures.

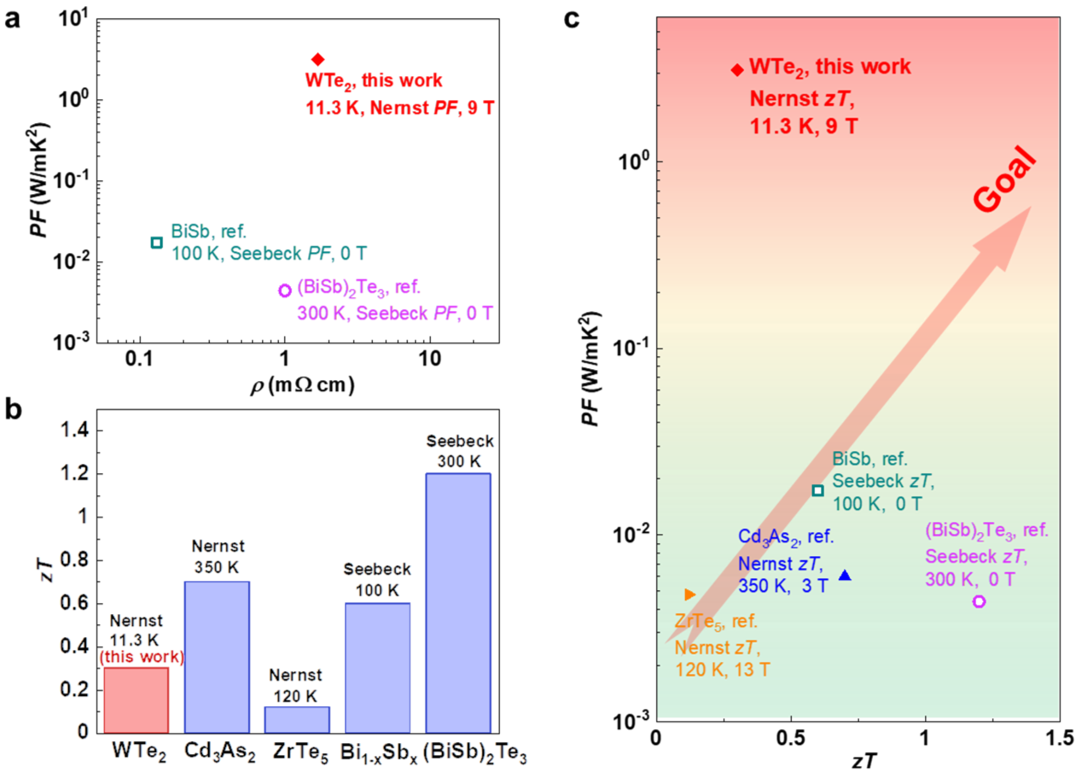

Supplementary Figure 5. Thermoelectric performance. Comparison of (a) *PF* and resistivity, (b) *zT* values, and (c) *PF* and *zT* of WTe_2 to other topological semimetals and classic thermoelectric semiconductor including $Bi_{1-x}Sb_x$ and $(Bi,Sb)_2Te_3$.

The WTe_2 single crystal shows high thermal conductivity, as shown in Supplementary Fig. 6. In this case, there is a large room to decrease the thermal conductivity of WTe_2, therefore, strategies like alloying can be effective to enhance its thermoelectric performance.

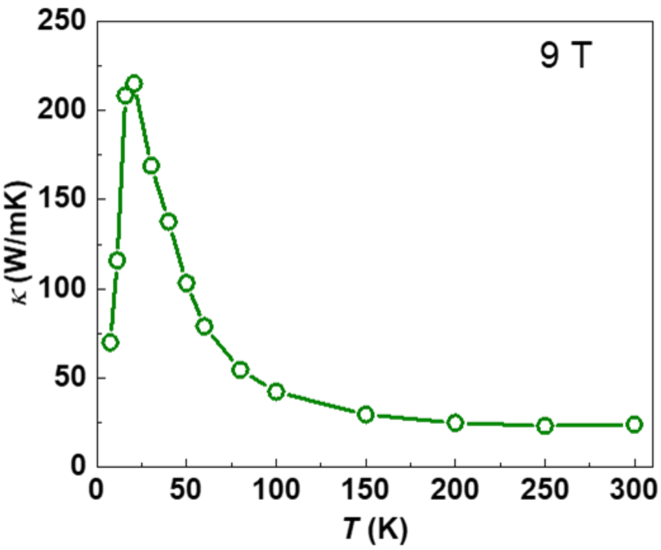

Supplementary Figure 6. Thermal conductivity. Temperature dependence of thermal conductivity.

Thermal transport properties' measurement setup

The Nernst thermopower, Ettingshausen temperature gradient, Seebeck coefficient, and thermal conductivity were measured in high vacuum in the PPMS by a standard four-contact, steady-state method. As shown in Supplementary Fig. 7, the sample was fixed on a piece of alumina attached on copper heater sink with a strain-gauge heater attached to the other end to apply a temperature gradient. Three sets of chromel-constantan thermocouples (in which two were used to measure the temperature difference along the temperature gradient direction, and one was used to measure the transverse temperature difference of the Ettingshausen signal) were mounted on the sample.

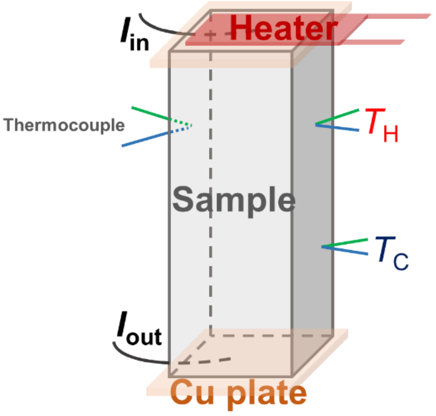

Supplementary Figure 7. Measurement setup. Schematic illustration of the sample setup for the thermal transport properties' measurement.

Supplementary References

1. W. M. Yim, A. Amith, Bi-Sb alloys for magneto-thermoelectric and thermomagnetic cooling, *Solid State Electron.* **15**, 1141 (1972).
2. B. Poudel, Q. Hao, Y. Ma, Y. Lan, A. Minnich, B. Yu, X. Yan, D. Wang, A. Muto, D.Vashaee, X. Chen, J. Liu, M. S. Dresselhaus, G. Chen, Z. Ren, High-thermoelectric performance of nanostructured bismuth antimony telluride bulk alloys, *Science* **320**, 634 (2008).
3. C. F. Kooi, R. B. Horst, K. F. Cuff, S. R. Hawkins, Theory of the longitudinally isothermal Ettingshausen Cooler, *J. Appl. Phys*. **34**, 1735 (1963).
4. P. Wang, C.-w. Cho, F. Tang, P. Wang, W. Zhang, M. He, G. Gu, X. Wu, Y. Shao, L. Zhang, Giant Nernst effect and field-enhanced transversal $z_N T$ in ZrTe5, *Phys. Rev. B* **103**, 045203 (2021).
5. J. Xiang, S. Hu, M. Lyu, W. Zhu, C. Ma, Z. Chen, F. Steglich, Ge. Chen, P. Sun, Large transverse thermoelectric figure of merit in a topological Dirac semimetal, *Sci. China. Phys. Mech.* **63**, 237011 (2020).
